# Specific Vicariance of Two Primeval Lowland Forest Lichen Indicators

**DOI:** 10.1007/s00267-017-0833-4

**Published:** 2017-02-15

**Authors:** Dariusz Kubiak, Piotr Osyczka

**Affiliations:** 1 Department of Mycology, University of Warmia and Mazury in Olsztyn, Oczapowskiego 1A, 10-719 Olsztyn, Poland; 2 Department of Polar Research and Documentation, Institute of Botany, Jagiellonian University, Kopernika 27, 31-501 Kraków, Poland

**Keywords:** Oak-hornbeam forest, Lichen biota, Lichen conservation, Environment evaluation, Host-tree, Habitat requirements

## Abstract

To date, the lichens *Chrysothrix candelaris* and *Varicellaria hemisphaerica* have been classified as accurate primeval lowland forest indicators. Both inhabit particularly valuable remnants of oak-hornbeam forests in Europe, but tend toward a specific kind of vicariance on a local scale. The present study was undertaken to determine habitat factors responsible for this phenomenon and verify the indicative and conservation value of these lichens. The main spatial and climatic parameters that, along with forest structure, potentially affect their distribution patterns and abundance were analysed in four complexes with typical oak-hornbeam stands in NE Poland. Fifty plots of 400 m^2^ each were chosen for detailed examination of stand structure and epiphytic lichens directly associated with the indicators. The study showed that the localities of the two species barely overlap within the same forest community in a relatively small geographical area. The occurrence of *Chrysothrix candelaris* depends basically only on microhabitat space provided by old oaks and its role as an indicator of the ecological continuity of habitat is limited. *Varicellaria hemisphaerica* is not tree specific but a sufficiently high moisture of habitat is essential for the species and it requires forests with high proportion of deciduous trees in a wide landscape scale. Local landscape-level habitat continuity is more important for this species than the current age of forest stand. Regardless of the indicative value, localities of both lichens within oak-hornbeam forests deserve the special protection status since they form unique assemblages of exclusive epiphytes, including those with high conservation value.

## Introduction

The spared remnants of natural deciduous or mixed forests in Europe represent a typical zonal formation constituting the dominant type of potential vegetation over large areas of the continent (Ellenberg 1988). Contrary to commercial forests, used primarily as a source of wood, deciduous forests have high environmental value, as they are characterised by great diversity of tree species and constitute important refuges for various organisms (Faliński and Mułenko 1995; Barbier et al. 2008).

Lichens are a conspicuous component, occupying the entire space of forest communities (Coxson and Nadkarni 1995; Sillett and Antoine 2004; Seaward 2008; Ellis 2012). While they occur in various ecological groups, epiphytes clearly dominate in deciduous and mixed forests of the temperate zone. Many lichens are closely related to a specific habitat and thus may possess indicative value (e.g., Nimis and Martellos 2001). Both external and internal factors, e.g., climate, landscape form, composition and maturity of trees, and biological interactions (Leppik and Jüriado 2008; Moning et al. 2009; Marini et al. 2011; Hauck et al. 2013; Nascimbene et al. 2013), affect lichen diversity in forest complexes. The relative effect of these factors on lichen biota development is difficult to assess (Pinho et al. 2008; Giordani and Brunialti 2015). Moreover, local climatic fluctuations, elevation, and/or land-use intensity drive the local specificity of lichen diversity (Loppi et al. 1997; Giordani 2006; Wolseley et al. 2006; Giordani and Incerti 2008). Generally, old-growth, least-affected lowland deciduous forests are characterised by a high level of lichen diversity, and many rare species are associated with old trees (Hyvärinen et al. 1992; Price and Hochachka 2001; Fritz et al. 2008a, b; Ranius et al. 2008a; Nascimbene et al. 2010a; Bartels and Chen 2012, 2014).

Numerous endangered epiphytic lichens are stenotopic and specially adapted to certain habitat types (Bartels and Chen 2015). These species may be a signal determinant of the natural condition of the forest environment (McCune 2000). The use of a limited number of bioindicators is an alternative and practical approach in environmental assessments (Nitare 2000). The great advantages of such an approach are a short study period, simplicity, and low cost (Will-Wolf et al. 2002; Gao et al. 2015). However, detailed data on the distribution and habitat requirements of lichen indicators are required for correct interpretation of field observations and for appropriate conservation actions and policies (Löhmus and Löhmus 2009; Juriado et al. 2012; Jönsson et al. 2016). Some species may have special conservation value, and thus their protection entails that of naturally co-occurring species and entire biocoenoses (Nilsson et al. 1995; Roberge and Angelstam 2004; Scheidegger and Werth 2009; Ivanowa 2015). Therefore, attention should be also paid to lichen biota directly associated with a particular indicator (Nilsson et al. 1995; Johansson and Gustafsson 2001; Nordén et al. 2007; Nascimbene et al. 2010b).

The impact of various factors on the local occurrence of two epiphytic lichens, *Chrysothrix candelaris* (L.) J. R. Laundon and *Varicellaria hemisphaerica* (Flörke) I. Schmitt & Lumbsch, within one type of forest community was examined in this study. These species are commonly used as suitable primeval lowland forest indicators (Coppins and Coppins 2002; Czyżewska and Cieśliński 2003; Motiejūnaitė et al. 2004) and are considered typical inhabitants of forests of above-average biodiversity (Andersson and Kriukelis 2002; Ek et al. 2002). The two species are morphologically distinctive and easy to identify in the field, even by non-specialists; therefore they have a wide practical application in field evaluations. Nevertheless, the lack of parallel contributions of *C. candelaris* and *V. hemisphaerica* in the epiphytic biota of some of the best-preserved European lowland forests is a mystery. It is difficult to find a study on lichens of old-growth lowland forest which does not report the presence of one of these species, yet the simultaneous existence of both populations in similar abundance within a single forest complex is observed only sporadically (Cieśliński and Tobolewski 1988; Zalewska 2012; Malíček and Palice 2013; Kubiak et al. 2016). Therefore, our research was aimed at answering the following questions: (1) Does the current structure of a forest stand affect the distribution of the examined species? If so, to what extent? (2) If so, what kinds of forest stands constitute optimal habitats for these species? (3) If not, are there other factors responsible for their distribution patterns? (4) Are specific assemblages of lichens directly associated with these species due to similar habitat requirements?

These questions seem particularly crucial in the context of the practical use of these lichens as environmental indicators. Moreover, we hypothesise that: (1) the distribution patterns of the examined species are not accidental and result from their narrow ecological amplitude; (2) their vicariance is caused by differences in forest stand structure; (3) both lichen indicators are leading representatives of unique assemblages of species with similar habitat requirements.

## Materials and Methods

### Study Area

**Fig. 1:**
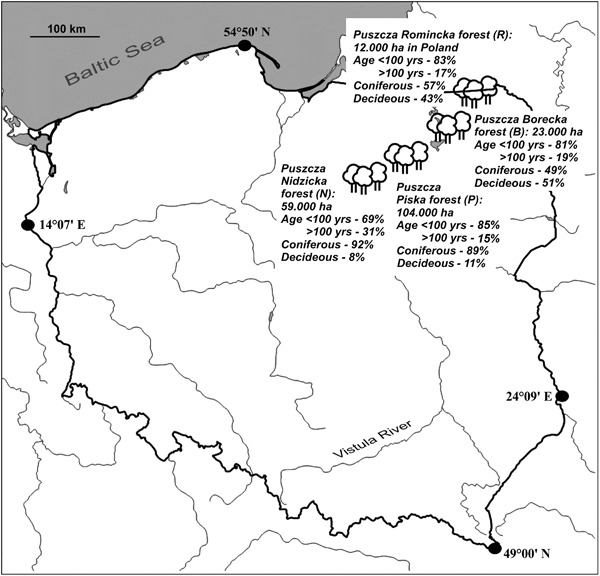
Location of the studied forest complexes in Poland. Total areas of the complexes and general coverage participations of forest stands within the complexes in relation to age (less than 100 years : over 100 years) and kinds of trees (coniferous : deciduous) are provided

The study was conducted in the Masurian Lakeland (NE Poland), almost one-third of which is covered by forests with a high degree of biodiversity (Faliński 1998). This region constitutes a natural corridor constituting an important environmental communication link in Central Europe (CORINE biotopes 2016). Field studies were carried out in four large forest complexes, in many parts of which oak-hornbeam stands have been preserved in their natural form to the present day: the Puszcza Nidzicka f. (abbr. N), Puszcza Piska f. (abbr. P), Puszcza Borecka f. (abbr. B), and Puszcza Romincka f. (abbr. R) (Fig. 1). The topography of the region is very diverse, as the landscape was formed during the Pomeranian phase of the last Vistula River glaciation (ca 15,200 BP) (Kozarski and Nowaczyk 1999). Denivelations are considerable, reaching as much as 120–140 m a.s.l; the highest point is almost 300 m a.s.l. This region is characterised by zonal climatic variability, with clashes of oceanic, continental, and boreal climate types (Kondracki 1972). Generally, the forest complexes situated further to the north-east, due to the greater impact of arctic air masses and relatively high elevation, experience a colder and more humid climate than those in the south-west. The vegetation period is relatively short, lasting between 160 to 190 days (Jutrzenka-Trzebiatowski 1999). As is apparent from the coverage maps of the Natura 2000 Special Area of Conservation (Interactive Map 2016), the B and R complexes are more compact and their state of preservation is considered more natural than that of the N and P complexes.

### Target Lichen Indicators

*Chrysothrix candelaris* (Online Resource 1) is identified by its bright yellow, diffuse, leprose thallus consisting of minute granules. The sterile form predominates in this species (Fletcher and Purvis 2009). *Varicellaria hemisphaerica* (Online Resource 1) forms an always sterile, rather thick, pale bluish-grey thallus with a usually broad margin and conspicuous, paler or concolourous, convex soralia (Chambers et al. 2009). Both species reproduce primarily by means of vegetative propagules (soredia); thus they likely possess similar dispersion abilities. The forest complexes under consideration are within the potential distribution range of *C. candelaris* and *V*. *hemisphaerica*; these species also occur in neighbouring countries (e.g., Litterski 1999; Prigodina Lukošienė and Naujalis 2009; Motiejūnaitė and Prigodina Lukošienė 2010). Generally, the species are characterised by low frequency in Central European forests (Dietrich and Scheidegger 1996; Svoboda et al. 2011) and are considered endangered or threatened in many countries (Piterāns 1996; Scheidegger and Clerc 2002; Cieśliński et al. 2006; Liška et al. 2008). They are sensitive to global environmental risks, although the eutrophication tolerance of *C. candelaris* is greater than was previously assumed (Pinho et al. 2011).

### Sampling Design and Data Collection

The field study was conducted in plots within a single community of a typical old oak-hornbeam forest corresponding to *Tilio-Carpinetum* Tracz. (see Faliński 1986). Initially, sixty-seven plots (N–20, P–20, B–17, R–10) of 400 m^2^ each inside the forests, i.e., at a distance of not less than 50 m from the edge of a uniform patch and at least 200 m from the forest line, were randomly selected (see also Friedel et al. 2006; Boch et al. 2013). The definition of a specific habitat as fresh broadleaved forest or fresh mixed deciduous forest, according to forest typology (Forest Data Bank 2016), was the basic criterion for the selection of the study plots. The second criterion was the presence of mature forest stands aged over 100 years. The study plots were not adjacent; up to 2 study plots were analysed within the area of a single forest division (ca 20 ha). The epiphytic lichen biota was examined in terms of the presence of *C. candelaris* and *V. hemisphaerica*. Nearly 900 trees, each with a diameter greater than 10 cm, were inspected. The lichen indicators were found in 50 plots. These plots were taken for further detailed consideration. In order to define the stands, all trees were counted and the diameter at breast height (DBH) of each tree was measured in the study plots. Tree species richness in plots can be considered to represent microhabitat heterogeneity; DBH is the equivalent of an available microhabitat for epiphytic lichens. Subsequently, the detailed scrutiny of epiphytic lichen biota was performed for trees serving as hosts for *C. candelaris* and *V. hemisphaerica.* The lichens were recorded on the tree trunks at a height of 0–2 m from the ground and classified into five classes according to the percentage scale of coverage: (1) <1%; (2) 1–5%; (3) 5–12.5%; (4) 12.5–50%; (5) >50%.

### Lichen Species Identification

The lichens were identified in the field only in cases of taxonomically non-problematic specimens. Most individuals, however, were collected for precise identification based on an examination of their macromorphological and micromorphological and chemical features. The lichens’ secondary compounds were analysed using the standard thin-layer chromatography (TLC) method, according to Orange et al. (2001). The nomenclature of the lichen species follows the Index Fungorum (2016). The collected lichen specimens are housed in the OLTC herbarium.

### Other Data Collection

**Fig. 2:**
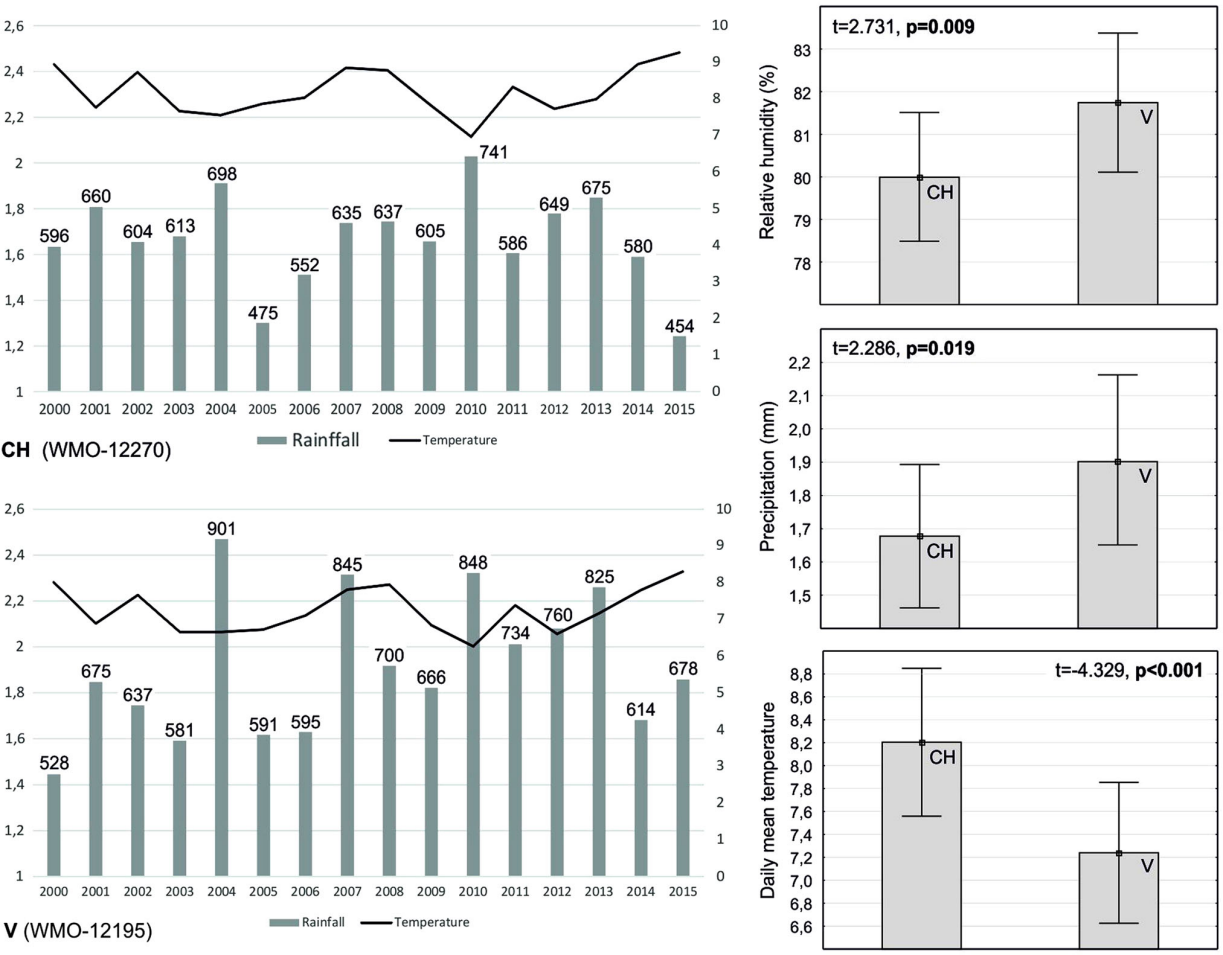
Obrothermic diagrams and main weather parameters corresponding to the plots with *Chrysothrix candelaris* (CH) and *Varicellaria hemisphaerica* (V). All graphs are prepared on the basis of daily reports from the years 2000–15 obtained from two selected meteorological stations (for details, see Materials and methods). The values above the bars present average annual rainfall. Mean values (points), standard deviation (whiskers), *t* and *p* values (significant are given in bold) are shown on small graphs (*n* = 5844)

The general coverage participation of coniferous and deciduous trees in the total areas of particular forest complexes (see Fig. 1), along with data on the general age of the forest stands directly corresponding to the particular plots, was derived from the official superintendence documentation; elevation of the plots was derived from detailed topographic maps. The procedure for determining the general age of a forest stand is based on the average age of the dominant tree species. Such an estimate, however, does not take into account the age of the youngest and oldest single trees. In order to characterise the basic weather conditions around the forest complexes, daily reports (relative humidity, precipitation, and mean temperature) from two selected meteorological stations from the beginning of the present century to the end of 2015 were downloaded and calculated, i.e., WMO index 12270 and 12195 (see Fig. 2). The first station is close to the N and P forests; the second is situated between the B and R forests.

### Statistical Analyses

The significance of differences between the *C. candelaris* and *V. hemisphaerica* plots in terms of forest stand structure and elevation were verified with multivariate Hotelling’s *T*-squared test. The same test was used to compare the data on weather conditions obtained from the meteorological stations. Multiple regression analysis was used to determine which of the independent variables, i.e., the age of the forest stand, number of trees, sum of DBH, and elevation, are the best predictors of *C. candelaris* and *V. hemisphaerica* abundance within their plots. The stepwise forward selection procedure was applied and the Pearson correlation coefficients calculated in advance to check whether any strong correlations (*r* > 0.90) exist between selected variables. Prior to the analysis, the coverage data were transformed, with the values from classes 2–5 being replaced by the average cover defined for these classes and the value 0.5 arbitrarily used for class 1. In addition to these analyses, differences between the plots with *C. candelaris* and those with *V. hemisphaerica* in terms of mean diameters of main host trees were tested with Student’s *t*-test. The Kolmogorov–Smirnov and Lilliefors tests were used to verify that the data were normally distributed; Levene’s test was applied to assess the equality of variances. Data which did not meet the assumptions of normality were Box–Cox transformed. When the aforementioned assumptions were not achieved, the non-parametric Mann–Whitney *U* test was used as an alternative. Permutational multivariate analysis of variance (PERMANOVA) was performed to test for differences between forest stands in terms of tree species composition (Anderson 2001); non-metric multidimensional scaling (NMDS) was used to obtain a diagram of the distribution of the plots. These analyses were based on the matrix of tree species abundance in particular study plots using the Bray–Curtis coefficient. NMDS and hierarchical clustering (Ward’s method) were applied to find the general pattern of similarities between lichen biotas (assemblages) directly associated with *C. candelaris* and with *V*. *hemisphaerica*. These analyses were based on the matrix of lichen species abundance using the Bray–Curtis coefficient. Detrended correspondence analysis (DCA) was performed to show the association of particular lichens with the main host trees for *C. candelaris* and *V*. *hemisphaerica.* To eliminate non-specific species, lichens that were common and abundant on all trees were excluded from the analysis. The Shannon diversity indexes, which were general and which took only main trees into account, were calculated for assemblages of lichens associated with *C. candelaris* and with *V. hemisphaerica*. The statistical calculations were performed using STATISTICA 12 and PAST 3.10 (Hammer et al. 2001).

## Results

### Vicariance of Target Lichen

The plots with *C. candelaris* were limited to the N (15) and P (10) forests, while almost all plots with *V. hemisphaerica* were located in the B (15) and R (8) forests. The only exceptions to this rule were two plots with *V*. *hemisphaerica* within the P forest; however, these were situated at the north-eastern end of the complex. The equal number of plots for both indicators is fortuitous.

### Characteristics of Forest Stands

**Fig. 3:**
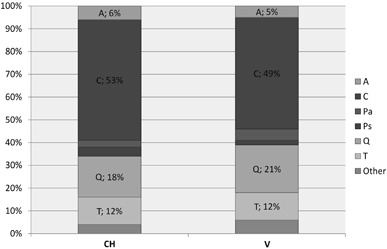
Forest stand structures expressed as the average proportion of tree species in the study plots where individuals of *Chrysothrix candelaris* (CH) and *Varicellaria hemisphaerica* (V) were recorded

The forest stands undergoing study consisted of 10 tree species: *Acer platanoides* (A), *Betula pendula* (B), *Carpinus betulus* (C), *Fraxinus excelsior* (F), *Picea abies* (Pa), *Pinus sylvestris* (Ps), *Populus tremula* (Pt), *Quercus robur* (Q), *Tilia cordata* (T), *Ulmus glabra* (U). For the average proportions of the main tree species, see Fig. 3. *Carpinus betulus* occurred throughout, *Quercus robur* and *Tilia cordata* in most of the studied plots, and *Acer platanoides* in a little over half of the plots; other tree species appeared sporadically. According to the PERMANOVA (*F* = 1.871, *p* = 0.121) and NMDS (Online Resource 2) analyses, tree species composition did not differ significantly between the plots with *C. candelaris* and those with *V. hemisphaerica*. Among four main tree components of the plots, only the mean diameter of oaks demonstrated a significant and strong correlation with the general age of forest stands. The mean DBH of other trees did not clearly correspond to the estimated age of the forests (Online Resource 3A).

### Factors Responsible for Differences between Plots and Affecting the Abundance of the Target Lichens

**Fig. 4:**
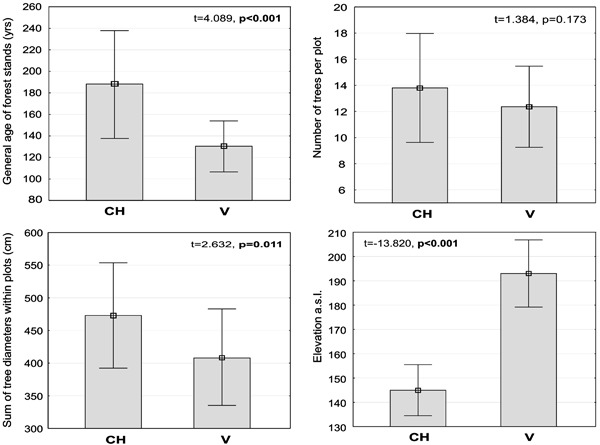
Characteristics of forest stands (age, tree density, sum of tree diameters) and elevation of the plots with *Chrysothrix candelaris* (CH; *n* = 25) and *Varicellaria hemisphaerica* (V; *n* = 25). Mean values (points), standard deviation (whiskers), *t* and *p* values (significant are given in bold) are shown on the graphs

Hotelling’s *T*-squared test revealed a highly significant combined effect of the independent variables on differences between the plots with *C. candelaris* and those with *V. hemisphaerica* (*T* ^2^ = 222.176; *p* < 0.05). The attributes differentiating the plots were related to the age of forest stands, sum of DBH, and elevation, with the last parameter exerting the most significant effect (Fig. 4); contrastingly, no significant difference in tree density was revealed. To generalise, the plots with *C. candelaris* are much more low-lying and represent older forest stands, usually composed of more venerable oaks (Fig. 4, Online Resource 3).

According to the multiple regression analysis, the only factor included in the model that positively and significantly (*p* < 0.05) influenced the abundance of *C. candelaris* was the age of the forest stand. In the case of *V*. *hemisphaerica*, three variables were entered in the model; of these, elevation and sum of DBH positively affect species abundance; however, only the effect of the former proved significant (*p* < 0.05). Tree density negatively affected the abundance of this lichen in the plots (*p* < 0.05).

### Climate

Annual precipitation in the twenty-first century varied considerably, from 454 to 902 mm, while the average annual temperature ranged between 6 and 9 °C. The weather patterns in the areas of the selected meteorological stations are substantially different. Comparison of the records revealed reliable differences between the main weather parameters (*T* ^2^ = 19.176; *p* < 0.05) and showed that temperatures in the WMO 12270 area were higher than in the WMO 12195 area, while relative humidity and precipitation were lower in the former. Total rainfall for the second station far exceeded 800 mm in some years (see Fig. 2).

### Host Tree Specificity of Target Lichens

**Fig. 5:**
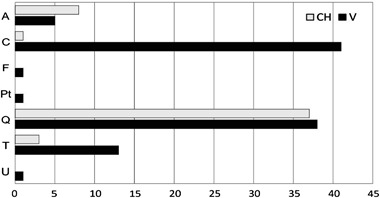
Host tree specificity of *Chrysothrix candelaris* (CH) and *Varicellaria hemisphaerica* (V) expressed as the total number of particular trees on which specimens of the lichens were recorded. Tree abbreviations: *Acer platanoides* (A), *Carpinus betulus* (C), *Fraxinus excelsior* (F), *Populus tremula* (Pt), *Quercus robur* (Q), *Tilia cordata* (T), *Ulmus glabra* (U)

Roughly half as many trees were inhabited by *C. candelaris* as by *V. hemisphaerica*; the totals were 49 and 100, respectively (for exact numbers in relation to particular tree species, see Fig. 5). Hornbeam was the most important host tree only for *V. hemisphaerica*. In particular, the largest hornbeams in the plots were readily inhabited by specimens of this lichen (Online Resource 3B). Oaks were the main host trees for both lichens, with nearly the same number of recorded trunks. However, oaks appeared to be essential for *C. candelaris* (Fig. 5); oaks overgrown by this lichen were characterised by significantly greater diameters than those inhabited by *V. hemisphaerica* (Online Resource 3B). The same applies to maples, though the difference was not significant according to the test. Maple was a preferred host tree for *C. candelaris*, linden for *V. hemisphaerica.* Single trunks of *Fraxinus*, *Populus*, and *Ulmus* were incidentally inhabited by *V. hemisphaerica* (Fig. 5).

### General Lichen Species Diversity

Altogether, 105 lichen species were recorded; of this pool, 74 occurred together with *C. candelaris* and 84 were associated with *V. hemisphaerica*. Only half of the species (to be exact, 53) turned out to be non-specific, co-occurring with both *C. candelaris* and *V. hemisphaerica*; the remainder were exclusive to assemblages of lichens associated with only one of these two species. Although a slightly greater number of exclusive species were associated with *V. hemisphaerica*, the two kinds of assemblages achieved similar average Shannon index values (see Table 1).

**Table 1:** The main properties of the lichen assemblages directly associated with *Chrysothrix candelaris* (Set 1) and *Varicellaria hemisphaerica* (Set 2)

Set 1 (for *Chrysothrix candelaris*)				Set 2 (for *Varicellaria hemisphaerica*)			
Total number of associated species: 74				Total number of associated species: 84			
Number of exclusive species: 21				Number of exclusive species: 31			
Shannon indexes				Shannon indexes			
General: 1.54–2.97 (2.38)				General: 1.69–3.2 (2.49)			
For A: 1.71–2.38 (2.49)				For A: 1.72–2.63 (2.26)			
For Q: 1.54–2.97 (2.34)				For C: 1.97–2.3 (2.67)			
				For Q: 1.69–3.03 (2.39)			
				For T: 1.69–2.87 (2.36)			
General characteristics:				General characteristics:			
Accepts a high proportion of coniferous trees in a wide landscape scale				Requires a high proportion of deciduous trees in a wide landscape scale			
Requires the presence of old deciduous trees, especially oaks, in a wide landscape scale				Consistency of forest stand with the habitat more important than its current age			
Prefers moderately dry habitats				Prefers moist habitats			
Exclusive species	Species abb.	F^a^	Host tree^b^	Exclusive species	Species abb.	F^a^	Host tree^b^
*Arthonia byssacea* (EN, +)	*Arth bys*	●●	A, Q	*Arthonia didyma* (EN, +)	*Arth did*	●●	C
*Arthonia muscigena*	*Arth mus*	●	A	*Arthonia radiata*	*Arth rad*	●●	C
*Bacidia arceutina* (EN, +)	*Baci arc*	●	C	*Arthonia vinosa* (+)	*Arth vin*	●●●●	C, Q, T, U
*Bacidia hemipolia* f. *pallida*	*Baci hem*	●	Q	*Arthothelium ruanum*	*Arth rua*	●●●	C, Q
*Calicium viride* (VU, +)	*Cali vir*	●●●●	A, Q, T	*Biatora ocelliformis* (VU, +)	*Biat oce*	●	C
*Caloplaca lucifuga*	*Calo luc*	●	Q	*Cladonia chlorophaea*	*Clad chl*	●	Q
*Chaenotheca brunneola* (EN, +)	*Chae bru*	●	Q	*Cladonia ochrochlora*	*Clad och*	●	A, Q
*Chaenotheca chrysocephala*	*Chae chr*	●●●●	A, Q, T	*Fuscidea pusilla*	*Fusc pus*	●	Q
*Chaenotheca furfuracea*	*Chae fur*	●●	Q	*Graphis scripta* sl	*Grap scr*	●●●●●	A, C, Q, T
*Chaenotheca phaeocephala* (EN)	*Chae pha*	●	A	*Lecanora carpinea*	*Leca car*	●●●	A, C, T
*Chaenotheca stemonea* (EN)	*Chae ste*	●●	Q	*Lecanora compallens*	*Leca com*	●	Q
*Chaenotheca trichialis*	*Chae tri*	●●●	A, C, Q	*Lecanora farinaria*	*Leca far*	●	C
***Chrysothrix candelaris*** **(CR, +)**	***Chry can***		**A, C, Q, T**	*Lecanora glabrata*	*Leca gla*	●●●	A, C
*Gyalecta truncignea* (EN)	*Gyal tru*	●	A	*Lecanora intumescens* (EN)	*Leca int*	●	C
*Lepraria vouauxii*	*Lepr vou*	●●	A, Q	*Lecanora pulicaris*	*Leca pul*	●●	C, T
*Lobaria pulmonaria* (EN, +)	*Loba pul*	●	Q	*Lecidea nylanderi*	*Leci nyl*	●	C
*Ochrolechia turneri*	*Ochr tur*	●●	A, Q	*Lecidella subviridis*	*Leci sub*	●●	C, Q, T
*Peltigera praetextata* (VU)	*Pelt pra*	●●	A, C	*Lepraria jackii*	*Lepr jac*	●	Q
*Phaeophyscia endophoenicea* (EN)	*Phae end*	●	C	*Mycoblastus fucatus*	*Myco fuc*	●●	C, T
*Physcia adscendens*	*Phys ads*	●	A	*Ochrolechia microstictoides*	*Ochr mic*	●	C, Q
*Physconia enteroxantha*	*Phys ent*	●●●	A, Q	*Opegrapha varia*	*Opeg var*	●	T
*Placynthiella icmalea*	*Plac icm*	●	Q	*Parmelia saxatilis*	*Parm sax*	●	C, Q
				*Parmelia submontana* (VU)	*Parm sub*	●	C
				*Pertusaria coronata* (VU, +)	*Pert cor*	●●●●	A, C, Q, T
				***Varicellaria hemisphaerica*** (**VU, +)**	***Pert hem***		**A, C, F, Pt, Q, T, U**
				*Pertusaria pertusa* (VU)	*Pert per*	●●●	C
				*Platismatia glauca*	*Plat gla*	●●●	C, F, Q
				*Porina aenea*	*Pori aen*	●●	C
				*Pyrenula nitida* (VU)	*Pyre ida*	●●●	C
				*Pyrenula nitidella* (EN, +)	*Pyre lla*	●●	C
				*Reichlingia leopoldii*	*Reic leo*	●	Q, T
				*Thelotrema lepadinum* (EN, +)	*Thel lep*	●	T

*CR* critically endangered, *EN* endangered, *VU* vulnerable, category in Red list of lichens in Poland, acc. to Cieśliński et al. (2006); + primeval lowland forest indicator, acc. to Motiejūnaitė et al. (2004). Bold font was used in order to indicate both analysed lichen species.

^a^ Frequency, percentage of tree trunks with *Chrysothrix candelaris*/*Varicellaria hemisphaerica*: ● <5%, ●● 5–10%, ●●● 11–25%, ●●●● 26–50%, ●●●●● >50%

^b^ *Tree abbreviations*: *Acer platanoides* (A), *Carpinus betulus* (C), *Fraxinus excelsior* (F), *Populus tremula* (Pt), *Quercus robur* (Q), *Tilia cordata* (T), *Ulmus glabra* (U)

### Lichen Assemblages

**Fig. 6:**
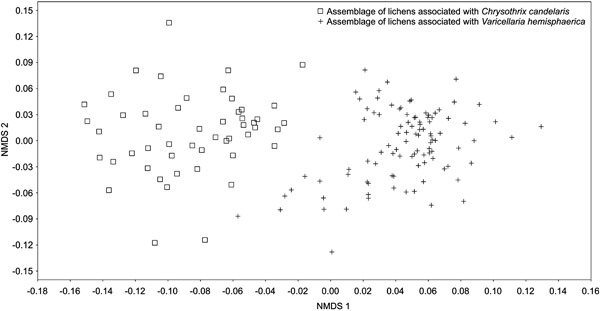
Non-metric multidimensional scaling (NMDS) ordination diagram showing the distribution of epiphytic lichen assemblages directly associated with *Chrysothrix candelaris* and *Varicellaria hemisphaerica*

NMDS ordination (Fig. 6) clearly separated the assemblages of lichens associated with *C. candelaris* (left side of the diagram) from those associated with *V. hemisphaerica* (right side); the two kinds of assemblages barely overlapped. Similarly, hierarchical clustering revealed two main distinct clusters (Online Resource 4). Cluster 1 included assemblages with *C. candelaris*, whereas cluster 2 comprised assemblages with *V. hemisphaerica*. The only disorder consisted of a small subcluster within the main cluster 1 which combined several assemblages associated with *C. candelaris* with others associated with *V. hemisphaerica*.

**Fig. 7:**
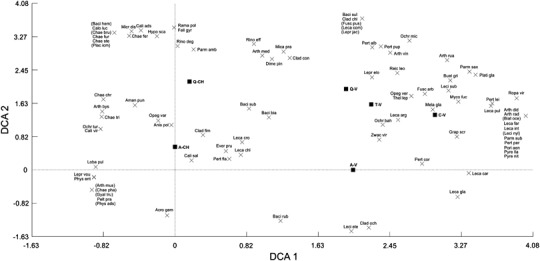
Detrended correspondence analysis (DCA) ordination diagram of the main host trees for *Chrysothrix candelaris* (A-CH, *Acer*; Q-CH, *Quercus*) and *Varicellaria hemisphaerica* (A-V, *Acer*; C-V, *Carpinus*; Q-V, *Quercus*; T-V, *Tilia*) and other lichen species associated both with these trees and with *C. candelaris*/*V. hemisphaerica*. Lichen species present as singletons are bracketed; for species abbreviations, as follows and see below Table 1. Abbreviations not included in Table 1: *Acro gem Acrocordia gemmata, Aman pun Amandinea punctata, Anis pol Anisomeridium polypori, Arth med Arthonia mediella, Baci bia Bacidia biatorina, Baci rub B. rubella*, *Baci sub* B. *subincompta, Baci sul B. sulphurella, Buel gri Buellia griseovirens, Cali ads Calicium adspersum, Cali sal C. salicinum, Chae fer Chaenotheca ferruginea, Clad con Cladonia coniocraea*, *Clad fim C. fimbriata, Dime pin Dimerella pineti*, *Ever pru Evernia prunastri, Fell gyr Fellhanera gyrophorica*, *Fusc arb Fuscidea arboricola*, *Hypo sca Hypocenomyce scalaris*, *Leca arg Lecanora argentata*, *Leca chl L. chlarotera*, *Leca cro Lecania croatica*, *Leci ele Lecidella eleaochroma*, *Lepr elo Lepraria elobata*, *Mela gla Melanelixia glabratula*, *Mica pra Micarea prasina agg*., *Micr dis Microcalicium disseminatum*, *Ochr bah Ochrolechia bahusiensis*, *Opeg ver Opegrapha vermicellifera*, *Parm amb Parmeliopsis ambigua*, *Pert alb Pertusaria albescens*, *Pert fla P. flavida*, *Pert lei P. leioplaca*, *Pert pup P. pupillaris*, *Pyre nit Pyrenula nitida*, *Rama pol Ramalina pollinaria*, *Rino deg Rinodina degeliana*, *Rino eff R. efflorescens*, *Ropa vir Ropalospora viridis*, *Zwac vir Zwackhia viridis*

The DCA diagram reflects the host specificity of associated lichen species in the context of trees inhabited by *C. candelaris* or *V. hemisphaerica* (Fig. 7). The eigenvalues of axes 1 and 2 were 0.602 and 0.274, respectively. The cumulative percentage of variance accounted by the first two axes equalled 54.2% (37.3% and 16.9% for the first and second axis, respectively). The DCA ordination diagram placed oak and maple with *C. candelaris* on the left side and grouped tree species with *V. hemisphaerica* on the right side. A link is evident between the strong host tree specificity of many lichens and their association with either *C. candelaris* or *V. hemisphaerica*. Several lichens in the central part of the diagram appear to have no special preference for a particular tree or indicator species.

## Discussion

Many lichens occupy specific micro-environmental niches and are sensitive to subtle environmental changes (Wirth 1992; Martinez et al. 2014). The results revealed that the occurrence of certain lichens defined as old-growth lowland forests indicators may be strongly influenced by their specific habitat requirements, which may rigorously determine their distribution patterns on a local scale. The localities of *C. candelaris* and *V. hemisphaerica* as a rule did not overlap, even though the examined plots represent the same type of forest stand.

### Forest Stand Structure

Many articles underline that tree species diversity and composition is one of the fundamental factors explaining epiphytic species distribution within forests (see Mežaka et al. 2008; Király et al. 2013). However, in this study it turned out that the studied plots could not be differentiated in terms of tree composition (Fig. 3 and Online Resource 2). Neither was there any significant difference in tree density. Thus it can be assumed that canopy closure and light availability were comparable in all of the plots (Fig. 4). The age of a stand is another key factor responsible for lichen biodiversity and composition (Fritz et al. 2008a, b; Nascimbene et al. 2010b). The oak-hornbeam forest stands inhabited by *C. candelaris* were generally older than those populated by *V. hemisphaerica* (Fig. 4). Additionally, the general age of forest stands was the only factor to positively affect the abundance of *C. candelaris* in the plots. It might simply be assumed that *C. candelaris* is attached to older forests. However, this species also appeared in the neighbouring 120-year-old mixed managed forests, provided that big oaks were present (Kubiak et al. 2016). Moreover, only the mean diameter of oaks in the plots, which was the essential host tree for *C. candelaris* (Fig. 5), significantly correlated—at least in this study (Online Resource 3a)—with the age of forest stands. Aged oaks, which are more likely to remain in old and unaffected oak-hornbeam stands, provide the microhabitat space necessary for *C. candelaris*. *Varicellaria hemisphaerica* has been frequently recorded in deciduous forests of uneven age, including those over two hundred years old (e.g., Fritz et al. 2008b). Therefore, we assume that the estimated fifty-year difference in the general age of current stands is not a direct and key factor regulating the occurrence of these species. It is very likely that the qualitative and age structure of the forest complex considered as a whole on a large-landscape scale has a greater influence on distribution patterns than the stand parameters directly characterising the examined plots. Although the coverage participation of forest stands over a hundred years old in the N and P complexes (approximately a third of the total) is somewhat higher than in B and R (less than one-fifth), the total participation of deciduous trees in the first two complexes falls within the vicinity of 10% (see Fig. 1). We believe that the vast total area of deciduous forest consistent with the habitat, along with its relatively low fragmentation, is of great importance for maintenance of *V. hemisphaerica*. On the other hand, *C. candelaris* showed no sensitivity to this general factor and even tended to colonise old mixed forests of anthropogenic origin (see Kubiak et al. 2016) or natural forests under strong human influence (Motiejūnaitė 2009).

### The Impact of Climatic Conditions

The occurrence of the examined lichen indicators is highly dependent on the geographical location of the forest complexes in which they grow. A relatively slight difference in location may be associated with quite different climate parameters. The studied complexes lie at similar distances from the coast of the Baltic Sea (about 100–150 km) and are not subject to the direct influence of a maritime climate. However, considering the study area, *C. candelaris* clearly prefers south-western complexes (the N and P forests), *V. hemisphaerica* north-eastern (the B and R forests). The complexes comfortable for *V. hemisphaerica* are located in an area with significantly higher relative humidity and precipitation as well as lower mean temperature compared to those preferred by *C. candelaris* (Fig. 2). The preference of *C. candelaris* for drier habitats and the attachment of *V. hemisphaerica* to moderately moist habitats are suggested based on an overview of the data in the literature (Fabiszewski and Szczepańska 2010). According to Wirth (2010), both lichens are characterised by similar ecological indicator values, although the moisture value is somewhat higher for *V. hemisphaerica.* The presence of *C. candelaris* in mixed forests with a high proportion of pine (see Kubiak et al. 2016) also indirectly suggests its low sensitivity to dehumidify of habitat (see also von Arx et al. 2012). The highest annual rainfalls in north-eastern Poland, which regularly exceed 700 mm, occur in the region of the R forest. These values are typical for the coastal zone of western Poland, where *V. hemisphaerica* is characterised by the highest frequency in the lowland part of the country within the beech distribution range (Fałtynowicz 1992). Mean annual precipitation is the most effective ecological predictor of the occurrence and diversity of many lichens (Svoboda et al. 2010; Merinero et al. 2014). In central Spain, *V. hemisphaerica* has been classified among the group of lichens very sensitive to the ‘edge of forest effect’ and strongly prefers the forest interior (Belinchón et al. 2007; Aragón et al. 2015). On the other hand, in Western European areas with very high humidity, this species appears in open habitats (Tønsberg 1992; Chambers et al. 2009). The dependence of *V. hemisphaerica* on climatic conditions and sufficient moisture of habitat may explain its low prevalence in the Białowieża Forest (Cieśliński 2003), one of the best preserved natural lowland forests in Europe, where suitable host trees certainly exist, but where annual rainfall and relative humidity are not as high as in the B and R forest complexes (Faliński 1986). Understanding the climatic sensitivity of species is a central theme in biodiversity conservation. Climate and response to climate change is one component among a large number of drivers which interact to control the occurrence of lichens (Lisewski and Ellis 2011).

### The Impact of Topography

Vertical distribution patterns of lichens have been usually investigated in mountain areas (Çobanoğlu and Sevgi 2009; Nascimbene and Marini 2015); the significance of elevation for lichen occurrence in the lowlands is rarely taken into consideration. However, the topography of some lowland areas may be characterised by highly diversified relief and great differences in elevation which often induce local climatic fluctuations. The average height above sea level of the B and R forests is higher by about 50 m than that of the N and P forests (Fig. 4). The elevation forces the convection of polar maritime air masses on the slopes of moraine hills and, furthermore, promotes condensation of water vapour in the air, consequently increasing the amount of rainfall (Huggett and Cheesman 2002). In the end, elevation turned out to be the only factor (rather than variables describing the general structure of stands) positively and significantly affecting *V*. *hemisphaerica* abundance within the plots. This is another point indicating the high moisture requirements of the species.

### Host Tree Specificity of Lichens

The presence of large old trees in the forest complex undoubtedly favours the occurrence of *C. candelaris*, which demonstrates strong dependence on oak trunks (Fig. 5 and Online Resource 3B). The age and bark structure of trees are among the most important factors determining the occurrence of certain epiphytes (Uliczka and Angelstam 1999; Johansson et al. 2007; Fritz et al. 2008a; Marmor et al. 2011). The average age of forest stands for plots with *C. candelaris* amounted to nearly 200 years; hence old and massive oaks have been preserved in large numbers in these forest complexes. The stands (including oaks) in the B and R forests are younger (Fig. 4); this may be one of the important causes for the negligible presence of *C. candelaris* in these complexes (see also Zalewska and Fałtynowicz 2004; Zalewska 2012; Forest Data Bank 2016). The same applies to maples, though *C. candelaris* shows a lesser preference for the trunks of this tree. The greater total sum of tree diameters and mean diameter of oaks and maples in the plots with *C. candelaris* compared to those with *V. hemisphaerica* (Fig. 4 and Online Resource 3B) suggests that the former species requires the provision by suitable host trees of a sufficiently large microhabitat surface. The positive impact on lichen species richness exerted by a high density of oaks in the immediate area in a larger landscape has been demonstrated (Ranius et al. 2008a, b; Paltto et al. 2010). Moreover, old oaks constitute almost the only harbour for *C. candelaris* in old managed forests planted in habitats typical of temperate deciduous forest (Kubiak et al. 2016). Numerous sources of lichen propagules and their proximity to suitable trees increase the probability that other trees will be colonised by epiphytic lichens (Johansson et al. 2009). In natural forests (e.g., the Białowieża Forest), where *C. candelaris* occurs abundantly, it sometimes grows on different trees (Cieśliński and Tobolewski 1988; Cieśliński 2003). Perhaps large and firmly established populations show greater tolerance in regard to host trees.

*Varicellaria hemisphaerica* is characterised by a much lower level of specialisation regarding species and age of host trees. Even though the number of plots with *C. candelaris* and *V. hemisphaerica* was the same, the latter was recorded on twice as many trees. *V. hemisphaerica* was recorded on almost all species of deciduous trees, with the exception of birch. However, hornbeam and oak constituted its main preferences (Fig. 5) and hornbeams with a large diameter were favoured by individuals (Online Resource 3b). In other deciduous forests, the thalli of *V. hemisphaerica* also readily overgrow beech bark, and this lichen can also be encountered on alders, hazels, and rowans (Fałtynowicz 1992; Cieśliński 2003). The bark of oaks, hornbeams, and lindens is fairly acidic (Barkman 1958; Jüriado et al. 2009) but the structure of these kinds of bark differs at various stages of the trees’ lives. It seems that a porous periderm structure, which appears only in old hornbeams, is an important factor for this lichen (Mežaka et al. 2008). In the Baltic countries, at the extremes of beech and hornbeam zone coverage, *V. hemisphaerica* inhabits mainly oaks (Prigodina Lukošienė and Naujalis 2006; Motiejūnaitė et al. 2008). The wide tolerance of *V. hemisphaerica* in relation to host trees may explain its frequency (twice that of *C. candelaris*) in the studied plots.

### Specific Assemblages of Lichens

Although our target species were *C. candelaris* and *V. hemisphaerica*, the study also concerned more than 100 accompanying lichen species. It can be stated that a large part of the lichen epiphytic biota characteristic of this type of forest community has been found to be directly associated with these indicators (cf. Cieśliński et al. 1995; Mežaka et al. 2008; Jüriado et al. 2009; Motiejūnaitė and Prigodina Lukošienė 2010; Svoboda et al. 2010, 2011). A slightly higher number of species recorded together with *V. hemisphaerica* results from the greater diversity of main host trees (Table 1 and Fig. 5). Both indicators co-create specific assemblages of epiphytic lichens (Fig. 6 and Online Resource 4) characterised by a high number of exclusive species (Table 1). These assemblages are similar in terms of species diversity but differ significantly in terms of species composition. Moreover, many lichens with high conservation value are integrated in both sets of lichens. Among the red-listed and endangered lichens in Poland (according to Cieśliński et al. 2006), *Arthonia byssacea* and *Calicium viride* (for the *C. candelaris* assemblage) and *Arthonia vinosa*, *Pertusaria coronata*, *P. pertusa, Pyrenula nitida*, and *P*. *nitidella* (for the *V. hemisphaerica* assemblage) were recorded most often. The great distinctness of the *C. candelaris* and *V. hemisphaerica* assemblages is symptomatic and indicates that their exclusive members exhibit similar habitat requirements. In addition, the intimacy of many accompanying species is frequently manifested in relation not only to *C. candelaris* and *V. hemisphaerica* but also to their main host trees (Fig. 7). Environmental evaluation should consider not only the separate occurrence of individual species but also the occurrence of sets of species forming specific communities. Assemblages of certain lichens are characterised by a high level of repeatability, whereas the appearance of individual species may be coincidental and insufficient for a proper diagnosis (see also Rola and Osyczka 2014).

### Conclusions: Management Implications and Conservation Value

Oak-hornbeam forests constitute the primary type of potential vegetation over large lowland areas of Europe, but at the same time are, due to agricultural expansion and the cultivation of wood-productive pines and spruces, the most endangered (Matuszkiewicz 2008). Therefore, this forest community has been included in the ecological network of special protected areas in Europe known as Natura 2000. One of the main objectives of this project (Council Directive 1992) is to preserve or restore valuable natural habitats. In practice, specifying the present condition of the habitat and determining the size of the protective area to be established for successful maintenance of the habitat are not simple tasks. Well-chosen lichen indicators can be of great assistance in such endeavours. Notwithstanding, the objective specification of lichen habitat requirements should be accomplished first, in order to define their actual indicative application.

Certain lichens are readily used to ascribe conservational value to old forest stands or natural woodlands (Nordén and Appelqvist 2001). Based on species composition, many biotas closely related to specific forest communities have been reported, and numerous lichens have been appointed as indicators of ‘ecological continuity’ (e.g., Rose 1974), ‘primeval (virgin) forest’ (e.g., Cieśliński et al. 1996), or ‘lowland old-growth forest’ (Czyżewska and Cieśliński 2003; Motiejūnaitė et al. 2004). Imprecise definitions of these concepts, ambiguous criteria for the selection of such indicators, and, consequently, interpretations of environmental information potentially provided by the occurrence of these lichens, often give rise to dispute (e.g., Nordén and Appelqvist 2001; Rolstad et al. 2002; Nordén et al. 2014). Specific local habitat parameters, the period required for the effective colonisation of suitable substrates, and natural disturbances of forest structure may in fact constitute key factors responsible for lichen biota development and the presence or absence of particular species (Kalwij et al. 2005). Our research showed that identification of potential habitat factors affecting the occurrence and abundance of particular indicators in the greatest possible detail is a desirable step towards the verification of their true bioindicative usefulness, especially in the context of a regional environmental evaluation.

*Varicellaria hemisphaerica* is not host-specific and readily inhabits trunks of various deciduous trees. A sufficiently high level of moisture in the habitat is essential for the species, which prefers forests with a high proportion of deciduous trees on a large-landscape scale. Habitat continuity on the level of the local landscape is more important for this species than the current age and structure of forest stands; thus, this lichen appears to be a good indicator of the ecological continuity of regional varieties of oak-hornbeam forest (see also Kubiak 2011, Kubiak and Łubek 2016). The opposite pattern is demonstrated by *Chrysothrix candelaris*. Among the many factors that determine the specificity of a given habitat, its occurrence basically depends on only one, i.e., the microhabitat space provided by old oaks. The lack of such trees in a given stand may seriously inhibit the lichen. Thus, its role as a general indicator of forest continuity in the context of a whole habitat is very limited.

Regardless of the indicative value, our study proved that localities within oak-hornbeam forests inhabited by both *C. candelaris* and *V. hemisphaerica* deserve special protection status. Many exclusive and endangered lichens with similar habitat requirements are associated with these two species (Table 1). Protecting their habitats may indirectly ensure the effective protection of many other lichens that make up coherent and stable epiphytic biotas. Moreover, the spontaneous restoration of deciduous forest areas consistent with the habitat, along with the associated lichen biota, is possible within a relatively short period. The occurrences of *C. candelaris* individuals on oaks in human-transformed mixed forests should be regarded as a positive phenomenon that may indicate that the process of natural regeneration is underway (see also Kubiak et al. 2016). Dense populations of *C. candelaris* and *V. hemisphaerica* may be a useful environmental tool for the designation of protected areas as ‘forests possessing unique environmental value’, according to the criteria of the High Conservation Value Forests programme HCVF (WWW 2007).

## Electronic supplementary material

Supplementary Online Resources
