# Supplementary material for: Specific Vicariance of Two Primeval Lowland Forest Lichen Indicators
Source: Environ Manage. 2017 Feb 15;59(6):966–81. doi: 10.1007/s00267-017-0833-4 (PMC5409926; doi:10.1007/s00267-017-0833-4)

Supplementary materials

Specific vicariance of two primeval lowland forest lichen indicators

Dariusz Kubiak, Piotr Osyczka

Online Resource 1. Epiphytic lichens *Chrysothrix candelaris* (on the left) and *Varicellaria hemisphaerica* (on the right) – old-growth lowland forest indicators. Scale bars = 1 cm.

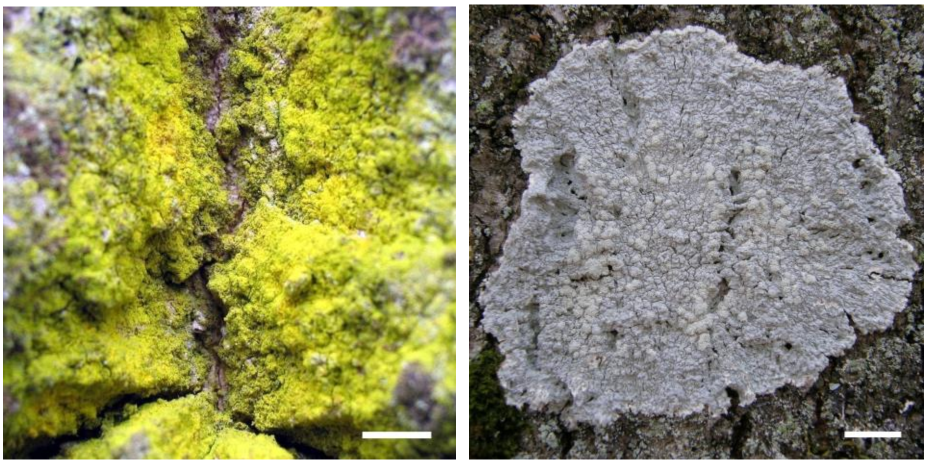

Online Resource 2. Non-metric multidimensional scaling (NMDS) ordination diagram showing the distribution of study plots (in terms of tree species composition) where individuals of *Chrysothrix candelaris* and *Varicellaria hemisphaerica* were recorded.

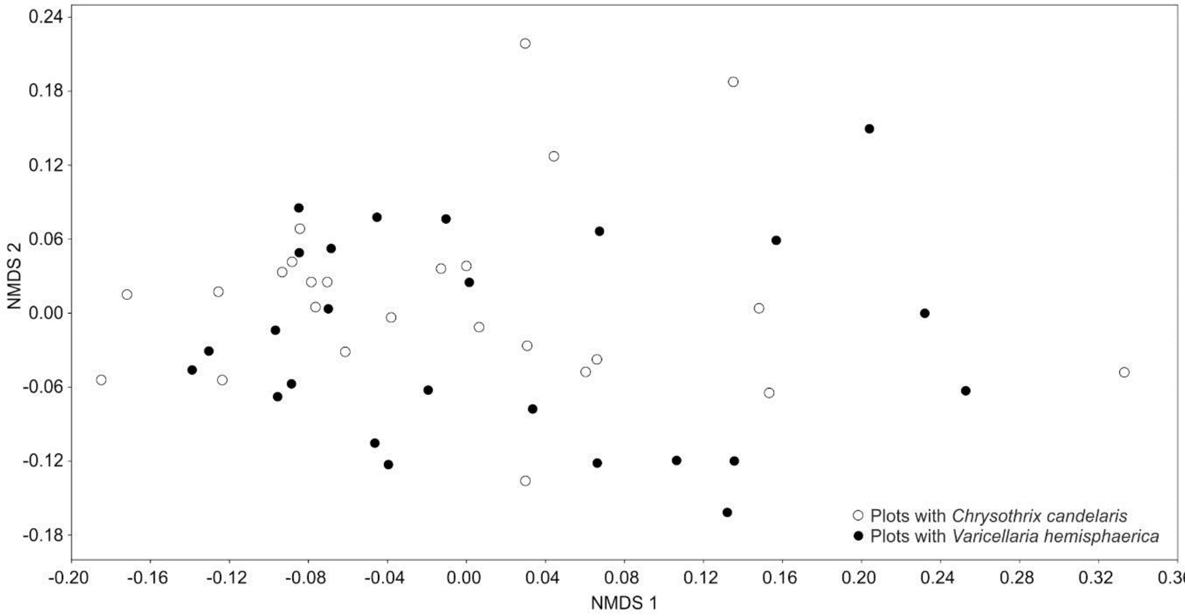

Online Resource 3. A – Scatterplots showing the relationships between the mean diameter of trees in the *Chrysothrix candelaris* and *Varicellaria hemisphaerica* plots and the general age of forest stands; significant correlation, revealed only for oak, with the Pearson coefficient (R) is presented. **B** – Comparison of mean diameters (DBH) of main trees calculated from the plots where individuals of *Chrysothrix candelaris* (CH) and *Varicellaria hemisphaerica* (V) were recoded. Both all trees within the plots (CH(T), V(T)) as well as only those occupied by lichen indicators (CH(L), V(L)) are considered. Mean values (points), standard deviation (whiskers) and the results of Student's t-test or Mann-Whitney U test are shown on graphs; significant *p* values are bolded.

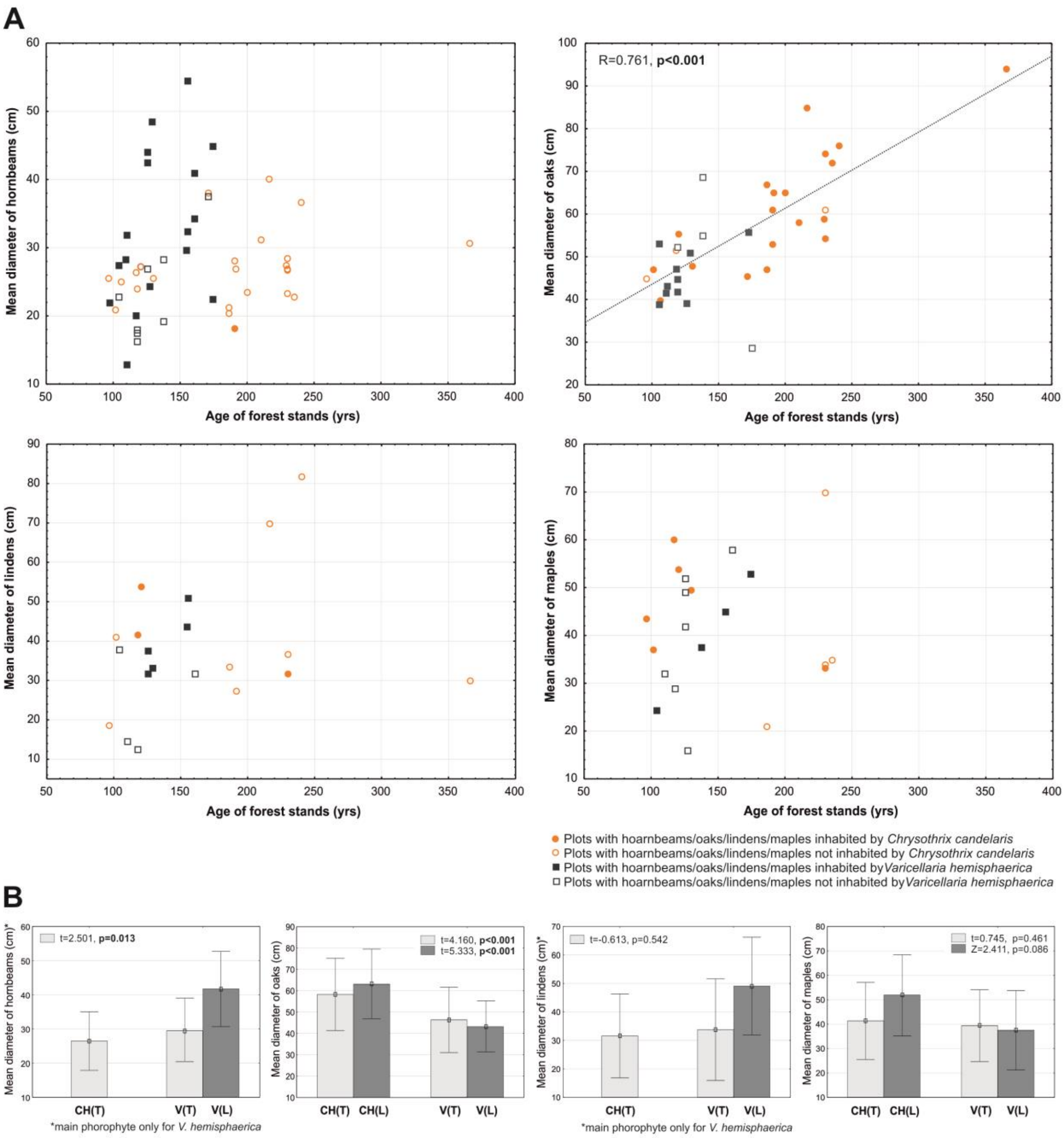

Online Resource 4. The dendrogram (Ward's method, Bray-Curtis coefficient) showing the similarities between epiphytic lichen assemblages directly associated with *Chrysothrix candelaris* and *Varicellaria hemisphaerica*.

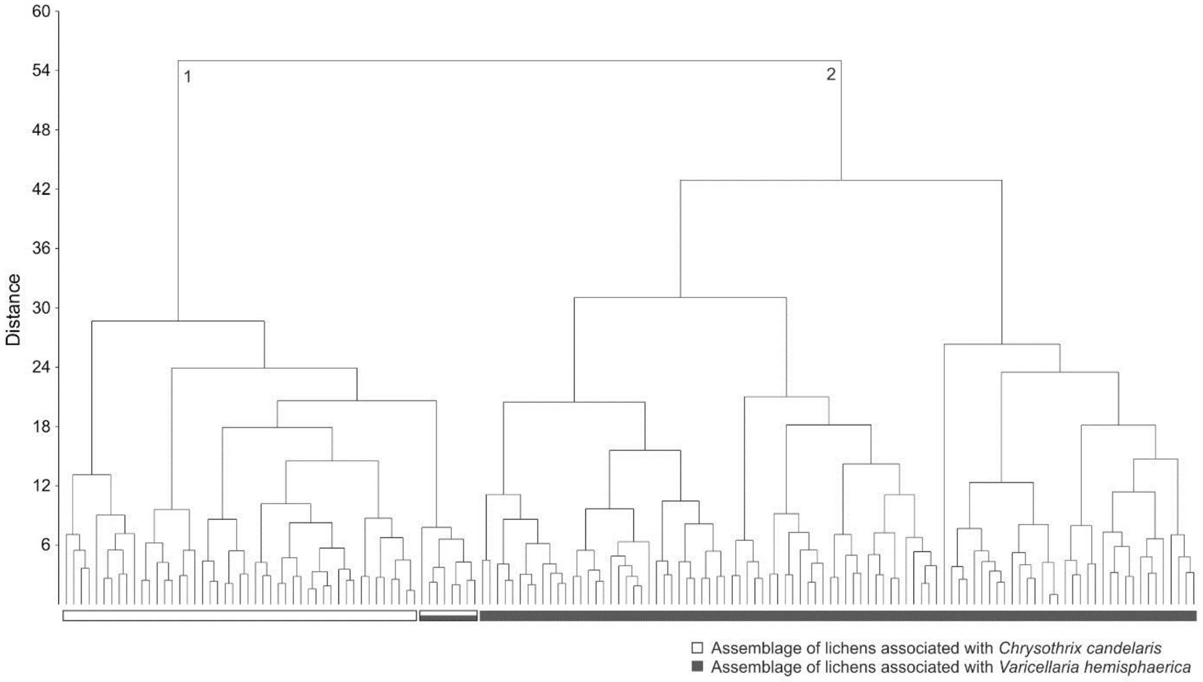

Supplement: Supplementary file 1 — Supplementary Online Resources [file 267_2017_833_MOESM1_ESM.pdf]
